# Perceptual asymmetries and handedness: a neglected link?

**DOI:** 10.3389/fpsyg.2014.00163

**Published:** 2014-02-28

**Authors:** Daniele Marzoli, Giulia Prete, Luca Tommasi

**Affiliations:** ^1^Department of Psychological Sciences, Humanities and Territory, University of ChietiChieti, Italy; ^2^Department of Neuroscience and Imaging, University of ChietiChieti, Italy

**Keywords:** perceptual and attentional asymmetries, handedness, left face bias, face, body

## Abstract

Healthy individuals tend to weigh in more the left than the right side of visual space in a variety of contexts, ranging from pseudoneglect to perceptual asymmetries for faces. Among the common explanations proposed for the attentional and perceptual advantages of the left visual field, a link with the prevalence of right-handedness in humans has never been suggested, although some evidence seems to converge in favor of a bias of spatial attention toward the region most likely coincident with another person’s right hand during a face-to-face interaction. Such a bias might imply an increased efficiency in monitoring both communicative and aggressive acts, the right limb being more used than the left in both types of behavior. Although attentional and perceptual asymmetries could be linked to right-handedness at the level of phylogeny because of the evolutionarily advantage of directing attention toward the region where others’ dominant hand usually operates, it is also legitimate to question whether, at the ontogenetic level, frequent exposure to right-handed individuals may foster leftward biases. These views are discussed in the light of extant literature, and a number of tests are proposed in order to assess our hypotheses.

As largely shown by past research, healthy individuals tend to weigh in more the left than the right side of visual space in a variety of contexts, ranging from pseudoneglect (for a review, see Jewell and McCourt, 2000) to perceptual and attentional asymmetries for faces (e.g., Burt and Perrett, 1997; Hsiao and Cottrell, 2008). Among the common explanations provided for the existence of such attentional and perceptual advantages of the left visual field, including hemispheric asymmetries for processing faces (Bentin et al., 1996; Kanwisher et al., 1997; Yovel et al., 2008; Prete et al., 2013), spatial information (Mesulam, 1981; Corbetta and Shulman, 2002), and social information (Brancucci et al., 2009), a possible functional link between leftward biases and the prevalence of right-handedness in human population has never been proposed, beyond a bulk of correlational studies. In fact, while it is controversial whether the right-hemispheric specialization for face processing drives the leftward bias for faces or vice versa (Dundas et al., 2012b), no one has attempted to relate such a bias to the potential advantage of better monitoring others’ dominant hand. In fact, it should be noted that the region of a person’s right hand and limb, with respect to an observer facing that person (assuming a face-to-face interaction), falls in the observer’s left field of view, so that any leftward bias would drive the focus of attention on the most active side of others’ body.

In other words, it could be not by chance that the location in space of others’ right hand, from the point of view of the observer, coincides with the well-known leftward bias of spatial attention, usually indicated as pseudoneglect (Bowers and Heilman, 1980; Jewell and McCourt, 2000). This is a natural property of attention whereby the left side of visual space is more relevant than the right side, as witnessed by the more frequent leftward errors made in bisection tasks (e.g., dividing a line or a rod into two halves), or by the fact that judgments on brightness, numerosity, and size are similarly skewed in favor of the left hemispace. A similar advantage for the left side is also observed in face perception, consisting in a preferential reliance upon the features on the left side of an observed face, when one has to make judgments about gender, attractiveness, age, and emotional expression conveyed by that face (Burt and Perrett, 1997). This is also reflected in the higher frequency of eye movements directed to the left side of the face (Butler et al., 2005). Given their biological relevance and their indivisibility from body – and thus from arms – in ecological settings, faces offer the best subject of inquiry to test our hypothesis.

## WHICH ARE THE POTENTIAL ADVANTAGES OF LEFTWARD ASYMMETRIES?

We believe that a bias of spatial attention toward the region most likely coincident with others’ right hand might have a deeply rooted justification in the communicative advantage conferred by attending to the limb most frequently used in gesturing, above all by right-handers and during speech (Kimura, 1973a,b; Dalby et al., 1980; Lavergne and Kimura, 1987; Saucier and Elias, 2001). Furthermore, the left-sided attentional bias might ensure a more efficient monitoring of aggressive behavior, the right limb being more used than the left also in violent actions (Coren and Porac, 1977). The other side of the coin is that a reduced monitoring of the right side of space – the space in which falls the dominant hand of encountered left-handed individuals – could contribute to the “surprise effect” at the basis of the left-handers’ advantage in fighting and sports (Raymond et al., 1996).

Clearly, this line of argument holds for right-handed subjects in the role of observers, and to some extent one would predict that it should also hold for left-handed observers, at least on the basis of visual experience. For example, Hagemann (2009) found that the directions of tennis strokes performed with the right hand were easier to predict compared to those performed with the left hand, regardless of observers’ handedness. Similarly, Marzoli et al. (in preparation) observed that, when required to report the perceived orientation (front or back view) of pictures of ambiguous human silhouettes performing one-handed manual actions, both right- and left-handers perceived the figure more frequently in an orientation congruent with a movement performed with the right rather than the left hand. However, one should not forget the possible contribution of motor representations in shaping attentional biases. In fact, the left hemispatial bias for face processing usually observed in right-handers is absent (Jaynes, 1976; Heller and Levy, 1981; Roszkowski and Snelbecker, 1982; Hoptman and Levy, 1988) or weaker (Luh et al., 1994) in left-handers. Moreover, left-handers turn out to be less affected by a leftward pseudoneglect (Brodie and Dunn, 2005; see also the meta-analysis by Jewell and McCourt, 2000). In support of a role of hand-related motor representations, attention has been reported to be biased toward the right and left side of observed bodies, regardless of their spatial orientation, respectively, in right- and left-handers (Gardner and Potts, 2010; see also Zartor et al., 2010). Analogous effects of handedness have been reported by our own group for the imagination of others’ actions (Marzoli et al., 2011a,b, in preparation). Noteworthy, these results seem to be in line with our proposal of a link between well-known attentional and perceptual leftward biases and an attentional bias toward the right side of others’ body, such biases being affected in similar ways by handedness. However, we point out that viewing perspective seems to interact with motor experience as regards attentional asymmetries toward others’ body, yielding a specific pattern of results (Marzoli et al., 2011a): when an actor is imagined as seen from the front, right-handers’ attention is biased toward their own dominant hand (that is, toward the left from their own point of view), whereas left-handers’ attention is biased toward their own non-dominant hand (that is, toward the left from their own point of view) or not biased at all; when an actor is imagined as seen from the back, both right-handers’ and left-handers’ attention is biased toward their own dominant hand (that is, toward the left from left-handers’ point of view and toward the right from right-handers’ point of view). Likewise, handedness does not affect perceptual and attentional asymmetries in the same direction in all tasks: form recognition and dot localization do not elicit any visual field difference between right- and left-handers, whereas letter recognition is performed better in the right visual field by right-handers, but not by left-handers (Bryden, 1973).

## DEVELOPMENTAL TREND IN THE LEFT FACE BIAS: IS THERE A ROLE FOR EXPERIENCE?

In our opinion, the role of experience in the establishment of perceptual asymmetries in face processing deserves in-depth investigation. For example, the direction of reading and writing systems that characterize the various human cultures (left-to-right or right-to-left) has been called into cause as a possible factor modulating the leftward lateral bias for face exploration and attention (Vaid and Singh, 1989; Sakhuja et al., 1996; Heath et al., 2005; Megreya and Havard, 2011). Therefore, we want to highlight how, although attentional and perceptual asymmetries could be linked to right-handedness at the level of phylogeny – because of the evolutionarily adaptive advantage of directing attention toward the region of visual space where others’ dominant hand usually operates – it is also legitimate to question whether, at the ontogenetic level, frequent exposure to right-handed individuals may foster leftward biases.

In this regard, it should be stressed that, whereas the leftward bias in face perception is usually observed in children of about 5 years (e.g., Roszkowski and Snelbecker, 1982; Levine and Levy, 1986; Kolb et al., 1992; Failla et al., 2003; Workman et al., 2006; Aljuhanay et al., 2010; Taylor et al., 2012), it is often reported to increase with age and reach an adult-like level by the age of about 10 years (Chiang et al., 2000; Workman et al., 2006; Anes and Short, 2009; Balas and Moulson, 2011; Taylor et al., 2012; Watling and Bourne, 2013; for a review, see Watling et al., 2012). However, the use of different methods seems to provide data in favor of both earlier (e.g., eye tracking; Wheeler, 2010; Liu et al., 2011; Dundas et al., 2012b) and later (e.g., moving window technique; Birmingham et al., 2012) emergence of an appreciable leftward bias in face processing. Similarly, a developmental trend has been shown in studies on the right-hemispheric advantage for face processing (Reynolds and Jeeves, 1978), although it is not always observed, maybe because of procedural differences (Young and Ellis, 1976; Young and Bion, 1980). Further support to our proposal can be drawn from studies showing that a general leftward bias for both upright and inverted human faces, monkey faces, and objects in infancy becomes a specific leftward bias for upright human faces in adulthood (Guo et al., 2009), and that the increase in leftward bias is specific for human faces, which suggests its experience-dependent nature (Balas and Moulson, 2011). A leftward bias for attending human faces was also reported for laboratory-raised rhesus monkeys and domestic dogs (Guo et al., 2009; see Dahl et al., 2013 for congruent findings in chimpanzees). Interestingly, the bias was absent for monkey and dog faces in dogs, and we believe that the prolonged experience with right-handed humans could be a more plausible account for such a specificity compared to other interpretations (e.g., a right-hemispheric specialization for human but not dog faces in both humans and dogs). Although rhesus monkeys showed a leftward bias for both human and monkey faces, given that they were presented only with human and monkey faces but not with dog faces, it cannot be resolved whether such a result was due to their difficulty in differentiating between the two species, to their experience with right-handed monkeys and humans, or to a non-species-specific bias. However, there is some evidence of right-handedness at least in captive rhesus monkeys (Westergaard and Suomi, 1996), as well as in other primates such as chimpanzees, gorillas, and baboons (see Hopkins, 2006; Cochet and Byrne, 2013; Meguerditchian et al., 2013 for reviews). We point out that population-level right-handedness is observed more often in captive rather than wild primates, as well as for communicative gestures rather than non-communicative actions, which has been credited to interaction with humans (Cochet and Byrne, 2013; Meguerditchian et al., 2013). This could suggest a crucial role for social factors also in the emergence of the left face/left visual field bias observed during emotional processing in nonhuman primates (see Lindell, 2013 for a review). On the other hand, findings from animal studies should be considered with caution as regards the origin of the leftward bias for faces, given that several results are inconsistent with a crucial role of interaction with humans even in domestic animals. For example, Racca et al. (2012) used emotional faces of both dogs and humans and found a more complex pattern of results compared to those of Guo et al. (2009), dogs showing a left gaze bias for conspecific negative expressions, a right gaze bias for conspecific positive expressions and no bias for conspecific neutral expression, as well as a left gaze bias for human negative and neutral expressions and no bias for human positive expressions. Moreover, sheep exhibit a left visual field advantage for conspecific (Peirce et al., 2000) but not for human faces (Peirce et al., 2001). Domestic chicks with no visual experience of human eyes and gaze also show a left visual field preference for monitoring a human-like dummy mask (Rosa Salva et al., 2007), which shows that even the emergence of leftward biases for human faces can be completely independent from interaction with humans.

The idea that the frequent interaction with right-handed individuals might promote leftward biases is consistent not only with both the experience-expectant and the experience-dependent view of brain development (Greenough et al., 1987), but also with previous studies showing that experience can affect the lateralization of face processing (e.g., infant holding biases; Vervloed et al., 2011; reading habits; Vaid and Singh, 1989; Sakhuja et al., 1996; Heath et al., 2005; Megreya and Havard, 2011). However, the fact that eye tracking studies reveal that a left visual field bias during face observation emerges within 9–11 months (Wheeler, 2010; Liu et al., 2011; Dundas et al., 2012b) and the fact that the leftward bias becomes more specific for upright human faces with increasing age (Guo et al., 2009) indicate that reading habits cannot account for the emergence of the bias. On the other hand, the cumulative experience with right-handed individuals might be responsible for the leftward bias increasing and becoming more selective with age. Moreover, given that the number of interactions with partners other than the primary caregiver increases with time, it should be investigated whether children of left-handed mothers show a shift from a rightward bias to a leftward one over time (in this regard, see Wheeler, 2010, who observed that in children aged 3–6 months with a rightward bias, this decreased with age).

The developmental trend in right-hemispheric specialization for faces has been credited to a parallel increase in right-hemispheric specialization for configural processing (Anes and Short, 2009). However, if the leftward bias for face processing is linked to configural processing, it should be noted that body configural information might include the knowledge (in terms of both first-order relational information and structural information; Reed et al., 2006) that the dominant hand of humans is usually placed on their right side, which could explain why face inversion, which disrupts configural processing (Maurer et al., 2002), also disrupts the leftward bias/right-hemispheric dominance in face processing (Ellis and Shepherd, 1975; Leehey et al., 1978; Luh, 1998; Coolican et al., 2008; Anes and Short, 2009; Bourne, 2011). The link between the leftward bias/right-hemispheric dominance and configural processing of faces is further corroborated by their similar developmental trends, configural processing and face-inversion effects also reaching adult-like levels by the age of 10 years (Carey and Diamond, 1977; Diamond and Carey, 1977; Mondloch et al., 2002), as well as by the finding that face-inversion effects appear to be stronger in the left rather than the right visual field (Leehey et al., 1978). In this regard, it is noteworthy that individuals with autism, who exhibit impaired configural processing (Behrmann et al., 2006), are less affected by both the face inversion effect (Hobson et al., 1988; Tantam et al., 1989) and the leftward bias for face processing (Dundas et al., 2012a; Taylor et al., 2012; see also Dundas et al., 2012b).

Another factor reported to affect the leftward bias for faces is maternal preferred cradling side: adults whose mother had an atypical right-side preference for holding infants show a reduced left-bias for chimeric faces compared to adults whose mother had the typical left-side preference (Vervloed et al., 2011). Interestingly, the maternal cradling side is also related to children’s handedness, right-cradled infants having slightly higher odds of being left-handed at 19 months of age (Scola and Vauclair, 2010). Given that children seem to imitate handedness preferences of adults (Harkins and Michel, 1988; Harkins and Uẑgiris, 1991; Michel, 1992; Fagard and Lemoine, 2006), imitation could also account for the greater incidence of left-handedness among right-cradled children, both because left-handed mothers are more likely to cradle on the right side (Scola and Vauclair, 2010) and because holding the infant on one side should free the opposite hand for other tasks (Huheey, 1977; see Hopkins, 2004 for similar associations between cradling side and hand preferences of both mother and infant in nonhuman primates). However, a reduced attentional bias toward the right arm might also explain the smaller leftward bias for faces observed in left-handers and in right-cradled individuals. This hypothesis deserves particular attention, above all in the light of the fact that individuals with autism, who show deficits in action imitation (see Williams et al., 2004 for a review), also exhibit a reduced leftward bias for face processing (Dundas et al., 2012a; Taylor et al., 2012; see also Dundas et al., 2012b) and a higher proportion of non-right-handedness (e.g., Escalante-Mead et al., 2003), which seems not to be accounted for by parental handedness (Tsai, 1982).

## SPECIFITY vs. GENERALIZABILITY OF LEFTWARD BIASES

Some evidence indicates that adult humans exhibit a leftward bias for upright human faces, but not for several other classes of stimuli such as vases, landscapes, and fractals (Mertens et al., 1993; Leonards and Scott-Samuel, 2005). Leonards and Scott-Samuel (2005) proposed that the leftward bias might be specific to socially relevant stimuli, and this could be in line with studies suggesting that the more the emotional load of the stimuli or tasks, the greater the leftward bias for faces (Gallois et al., 1989; Coolican et al., 2008; Thompson et al., 2009). In line with this proposal, centrally presented gaze cues (i.e., social stimuli) facilitate the detection of spatially congruent targets presented in the left visual field (that is, the region of the observed person’s right hand during a face-to-face interaction) but not in the right visual field, whereas arrow cues (i.e., non-social stimuli) are effective for targets presented in both visual fields (Marotta et al., 2012; see also Greene and Zaidel, 2011).

The role of social relevance in the emergence of attentional asymmetries in favor of the left visual field is corroborated by a series of studies by Mogg and Bradley (1999, 2002) showing that threatening faces induced a greater attentional capture compared to happy and neutral faces when the faces were subliminally presented in the left but not in the right visual field, and that this effect was particularly apparent for more anxious individuals. A study by Field (2006) found a similar pattern of results, extending the leftward bias for threatening stimuli to a different population (children aged 7–9 years) and different stimuli (animals). Therefore, although the leftward bias/right hemispheric advantage could be more evident for faces, it is not exclusive of this class of stimuli, as also shown by studies generalizing the left visual field bias to photographs of houses and cars (Levine et al., 1984) and line drawings of common objects (Kim et al., 1990). Nonetheless, it is not unreasonable to hypothesize that more general attentional and perceptual asymmetries may arise from an initial leftward bias for faces and/or bodies. Specifically, given that human bodies and faces are the most ecologically relevant and likely the most recurrent stimuli people deal with in everyday life, the asymmetrical processing they elicit could generalize to some extent to other domains. This view would be consistent with the observation that handedness and sex seem to affect the left side bias for faces and other leftward asymmetries in similar ways: according to a meta-analysis of line bisection studies conducted by Jewell and McCourt (2000), in fact, males show a slightly larger pseudoneglect compared to females and right-handers show a slightly larger pseudoneglect compared to left-handers. Interestingly, this latter finding cannot be attributed to the mere use of the left hand, because the authors also mentioned a relative bias in the direction of the hand used to perform bisection, which is consistent with the activation-orientation theory of Kinsbourne (1970). On the contrary, the modulation of pseudoneglect by handedness could match the way in which one’s own motor representations seem to affect attentional asymmetries toward humans bodies observed from the front (e.g., Gardner and Potts, 2010; Marzoli et al., 2011a). Finally, the fact that pseudoneglect shows a developmental trend similar to that of the leftward bias for faces also suggests their related origin (Bradshaw et al., 1988; Dellatolas et al., 1996; Failla et al., 2003). However, it should be noted that a left-sided visuospatial bias has also been found in birds (Diekamp et al., 2005), and embryonic light stimulation has been invoked for its emergence (Chiandetti, 2011), which suggests that pseudoneglect could arise from causes other than the social ones.

## EMOTIONAL ASYMMETRIES

On the basis of the literature reviewed in the previous section, social stimuli, and emotional stimuli in particular, are more likely to induce attentional and perceptual asymmetries compared to non social stimuli. In this section, we attempt to conciliate the larger asymmetries observed for emotional stimuli with our main hypothesis. As recently stressed by Watling et al. (2012), future research should address the advantages of lateralization for emotion processing, as well as related gender differences. In this regard, a positive correlation has been observed between children’s left hemispatial advantage for emotion perception and their ability to understand emotional states in cartoon situations and in eyes (Workman et al., 2006), as well as in faces, although this was shown only in male children (Watling and Bourne, 2013). However, a recent study extended the positive correlation between left-lateralized processing and performance to the discrimination of both human and chimpanzee faces in both species (Dahl et al., 2013). These studies suggest a link between the lateralization of emotional processing and the understanding of others’ emotional/cognitive states, which is bolstered by their similar time course, theory of mind emerging by the age of 4 years and improving during childhood (Baron-Cohen, 1995). Moreover, the leftward bias for faces approaches adult-like levels by the age of 10 years (Chiang et al., 2000; Workman et al., 2006; Anes and Short, 2009; Taylor et al., 2012), just before children start to exhibit a preference for the left eye (from the observer’s viewpoint) during face scanning (Birmingham et al., 2012) and a patent improvement in their ability to interpret emotion from eyes (Tonks et al., 2007).

Therefore, one could wonder whether the advantage of the right-hemispheric specialization for emotion processing might lie in monitoring other’s emotional states and their subsequent actions within the same hemisphere, and whether leftward biases could be strengthened by the fact that interaction partners’ facial expressions and eye movements are constantly associated with their right-handed actions. This hypothesis deserves particular consideration, given that the leftward bias for emotion processing could appear counterintuitive, emotions being expressed more intensely on the left side of the face, which falls in the right visual field of the observer in a face-to-face interaction (Sackeim and Gur, 1978). On the other hand, there is some evidence that anger might be expressed more intensely on the right side of the face (Indersmitten and Gur, 2003) and that the leftward bias might be larger for anti-social emotions (and in particular for anger) than for pro-social emotions (Workman et al., 2000). Thus, the leftward bias appears to be less counterintuitive if one assumes that both bearing a particular sensitivity to the hemiface expressing more intense threat-related facial displays and directing attention toward the region containing the right arm of an angry individual could provide important ecological advantages. This could be particularly true during interactions among males, and we would like to point out that the leftward bias has been reported to be stronger in males than in females (Bourne, 2008; see also Godard and Fiori, 2010). Moreover, in males the leftward bias reaches its highest degree when they observe male faces expressing anger rather than male faces expressing the other five basic emotions or female faces expressing all basic emotions (Rahman and Anchassi, 2012). The uniqueness of anger among emotions has already been proposed by Indersmitten and Gur (2003; see Workman et al., 2000 for similar considerations), who stressed both its nature of evolutionarily important sign for action (its purpose is to prepare the organism for conflict) and its increased likelihood to be appreciated by the perceiver (its greater intensity on the right rather than the left hemiface enhances its impact on the hemisphere more dominant in emotion processing). In the same vein, it is not surprising that more anxious individuals exhibit a greater leftward bias compared to less anxious ones (Heller et al., 1995; Keller et al., 2000; Voelz et al., 2001; Bourne and Vladeanu, 2011), and therefore an interesting experimental question is whether the former also show greater attention toward the right limbs of human bodies compared to the latter.

We would like to remark that the advantages of lateralization for emotion processing discussed in this section are in agreement with previous suggestions (e.g., Vallortigara and Rogers, 2005) that (i) the lateralization of cerebral functions enhances cognitive capacity and efficiency (a positive correlation existing between the leftward bias for emotion perception and performance in emotion discrimination), and (ii) the alignment of the direction of behavioral asymmetries at the population level emerges, as an evolutionary stable strategy, under social pressures (the leftward bias for emotion processing being credited to the advantage of monitoring others’ emotional states and their dominant hand within the same hemisphere).

## COUPLING BETWEEN FACE AND BODY PROCESSING

Our proposal that the leftward bias for faces might be associated with a similar bias for bodies is supported by several analogies between face and body processing, including the importance of configural information, the inversion effect affecting both categories (e.g., Reed et al., 2003), and embodied experience, humans being able to move both faces and bodies (Slaughter et al., 2004). On the other hand, face and body representations are likely to differ at least to some extent (e.g., Soria Bauser et al., 2011). Moreover, although both face and body processing develop early in infancy, there is some evidence that face expertise may precede body expertise (Heron-Delaney et al., 2011; Slaughter et al., 2002). A possible account for such a differential development is that the earliest social experiences between infants and caregivers involve a face to face interaction, so that infants are exposed more often to faces than to whole bodies. Surely, face and body representations interact reciprocally (van de Riet and de Gelder, 2008; Yovel et al., 2010; Aviezer et al., 2012) and also induce similar responses (Tamietto et al., 2009).

At the neural level, the same area, the right fusiform gyrus, contains representations for both faces (Kanwisher et al., 1997) and bodies (Peelen and Downing, 2005). Although largely overlapping (Peelen and Downing, 2005), the fusiform face and body areas (FFA, FBA) turned out not to be identical (Schwarzlose et al., 2005; Peelen et al., 2006). Given that the magnitude of the asymmetry of the FFA strongly correlates with leftward asymmetries in face perception (Yovel et al., 2008), the existence of a similar association between FBA and perceptual and attentional asymmetries toward the right side of human bodies deserves investigation. Moreover, whereas the size and selectivity of the rFFA increase with age (Aylward et al., 2005; Golarai et al., 2007; Scherf et al., 2007; Peelen et al., 2009), matching the developmental trend of face-related configural processing and leftward bias, those of the rFBA do not differ between children and adults, this region not showing any development beyond the age of 7 years (Peelen et al., 2009). Thus, given that the age-related increase of the leftward bias for faces can be explained also in terms of the mere maturation of the biological substrate, it could be investigated whether an age-dependent increase in the attention allocated to the right side of human bodies exists and, if so, whether it pre-exists that observed for faces. According to Peelen et al. (2009), a possible account for the differential development of rFFA and rFBA is that young children, when not looking up, usually observe the bodies rather than the faces of older (and thus taller) individuals, whereas adults are more likely to observe the faces of other individuals. For the same reason, the dominant hand might be associated earlier to the right side of bodies rather than of faces, which could contribute to explain why the rFBA reaches adult size before the rFFA. The FFA is also more right-lateralized in right-handers than in left-handers (Willems et al., 2010), in line with the weaker left face bias observed in left-handers. Although less consistent, similar effects of handedness have been reported for the FBA (Willems et al., 2010), which could be linked to the weaker bias toward the right side of bodies observed in left-handers (Gardner and Potts, 2010; Marzoli et al., 2011a,b, 2013).

## CONCLUSION AND FUTURE DIRECTIONS

Although different adaptive reasons have been proposed for the evolution of human right-handedness (Cochet and Byrne, 2013), the adaptive functions of the left face bias, as well as, broadly speaking, perceptual, and attentional asymmetries, have not received the same consideration. The present article attempts to provide a contribution in this direction, suggesting several research questions. The first prediction derived from our hypothesis is that the intensities of the leftward bias for faces and for bodies should be correlated. The leftward bias for bodies could also be modulated by the same factors affecting the leftward bias for faces, such as maternal cradling preference, age, anxiety, emotional context (for example, the presentation of angry faces or voices should increase the bias), configural processing (the bias should be reduced by inversion), and so on.

Moreover, a major topic of investigation should be the effect of experience with right-handed individuals in inducing leftward biases (for both faces and bodies). For example, it could be expected that the bias would be stronger for faces and bodies of highly familiar right-handed individuals than for faces and bodies of unfamiliar individuals. In the same respect, the discovery that dogs show a selective left face bias for human faces (Guo et al., 2009) offers an interesting opportunity to investigate the role of experience also in a nonhuman species. Specifically, it could be tested whether the bias is weakened, or even reversed, in dogs that have interacted mainly with left-handed individuals (i.e., owners, breeders, trainers). Such a study would provide useful information on the contribution of sensory experience in the manifestation and perhaps even in the origin of a perceptual asymmetry whose existence is known since several decades in human beings, but that has recently been observed also in other species.

Finally, an important field of study could address the topic of leftward biases in individuals with autism, who exhibit deficits in social communication (Klin et al., 2003) and emotion recognition from both faces and bodies (Philip et al., 2010), as well as in inferring others’ complex mental states from faces and particularly by eyes (Baron-Cohen et al., 1997). These individuals are known to show impaired configural processing (Behrmann et al., 2006) and an absent (Dundas et al., 2012a) – or at least delayed (Taylor et al., 2012) – perceptual bias for the left side of faces. Given the link between face and body representations, it should be investigated whether in this population the reduced leftward bias for faces is coupled with a reduced leftward bias for bodies, just as a reduced face-inversion effect (Hobson et al., 1988; Tantam et al., 1989) is coupled with a reduced body inversion effect (Reed et al., 2007). Moreover, it would be interesting to examine whether action imitation deficits of individual with autism are positively related to non-right-handedness and negatively related to leftward biases toward faces and bodies. If so, a reduced attention toward the right side of human bodies could be responsible for the abnormal pattern of behavioral asymmetries in the autistic disorder, endorsing once again the role of body representations in social cognition.

## Conflict of Interest Statement

The authors declare that the research was conducted in the absence of any commercial or financial relationships that could be construed as a potential conflict of interest.

## References

[B1] Aljuhanay A. Milne E. Burt D. M. Pascalis O. (2010). Asymmetry in face processing during childhood measured with chimeric faces. *Laterality* 15 439–450 10.1080/13576500902972823 19548168

[B2] Anes M. D. Short L. A. (2009). Adult-like competence in perceptual encoding of facial configuration by the right hemisphere emerges after 10 years of age. *Perception* 38 333–342 10.1068/p6092 19485130

[B3] Aviezer H. Trope Y. Todorov A. (2012). Holistic person processing: faces with bodies tell the whole story. *J. Pers. Soc. Psychol.* 103 20–37 10.1037/a0027411 22352325

[B4] Aylward E. H. Park J. E. Field K. M. Parsons A. C. Richards T. L. Cramer S. C. (2005). Brain activation during face perception: evidence of a developmental change. *J. Cogn. Neurosci.* 17 308–319 10.1162/0898929053124884 15811242

[B5] Balas B. Moulson M. C. (2011). Developing a side bias for conspecific faces during childhood. *Dev. Psychol.* 47 1472–1478 10.1037/a0024494 21767012

[B6] Baron-Cohen S. (1995). *Mindblindness: An Essay on Autism and Theory of Mind*. Cambridge, MA: MIT Press

[B7] Baron-Cohen S. Wheelwright S. Joliffe T. (1997). Is there a “language of the eyes?” Evidence from normal adults, and adults with autism or Asperger syndrome. *Vis. Cogn.* 4 311–331 10.1080/713756761

[B8] Behrmann M. Avidan G. Leonard G. L. Kimchi R. Luna B. Humphreys K. (2006). Configural processing in autism and its relationship to face processing. *Neuropsychologia* 44 110–129 10.1016/j.neuropsychologia.2005.04.002 15907952

[B9] Bentin S. Allison T. Puce A. Perez E. McCarthy G. (1996). Electrophysiological studies of face perception in humans. *J. Cogn. Neurosci.* 8 551–565 10.1162/jocn.1996.8.6.551 20740065 PMC2927138

[B10] Birmingham E. Meixner T. Iarocci G. Kanan C. Smilek D. Tanaka J. W. (2012). The moving window technique: a window into developmental changes in attention during facial emotion recognition. *Child Dev.* 84 1407–1424 10.1111/cdev.12039 23252761

[B11] Bourne V. J. (2008). Examining the relationship between degree of handedness and degree of cerebral lateralization for processing facial emotion. *Neuropsychology* 22 350–356 10.1037/0894-4105.22.3.350 18444713

[B12] Bourne V. J. (2011). Examining the effects of inversion on lateralisation for processing facial emotion. *Cortex* 47 690–695 10.1016/j.cortex.2010.04.003 20541184

[B13] Bourne V. J. Vladeanu M. (2011). Lateralisation for processing facial emotion and anxiety: contrasting state, trait and social anxiety. *Neuropsychologia* 49 1343–1349 10.1016/j.neuropsychologia.2011.02.008 21315746

[B14] Bowers D. Heilman K. M. (1980). Pseudoneglect: effects of hemispace on a tactile line bisection task. *Neuropsychologia* 18 491–498 10.1016/0028-3932(80)90151-7 6777712

[B15] Bradshaw J. L. Spataro J. A. Harris M. Nettleton N. C. Bradshaw J. (1988). Crossing the midline by four to eight year old children. *Neuropsychologia* 26 221–235 10.1016/0028-3932(88)90076-0 3399040

[B16] Brancucci A. Lucci G. Mazzatenta A. Tommasi L. (2009). Asymmetries of the human social brain in the visual, auditory and chemical modalities. *Philos. Trans. R. Soc. Lond. B Biol. Sci. * 364 895–914 10.1098/rstb.2008.0279 19064350 PMC2666086

[B17] Brodie E. E. Dunn E. M. (2005). Visual line bisection in sinistrals and dextrals as a function of hemispace, hand, and scan direction. *Brain Cogn.* 58 149–156 10.1016/j.bandc.2004.09.019 15919545

[B18] Bryden M. P. (1973). Perceptual asymmetry in vision: relation to handedness, eyedness, and speech lateralization. *Cortex* 9 419–435 10.1016/S0010-9452(73)80041-3 4784699

[B19] Burt D. M. Perrett D. I. (1997). Perceptual asymmetries in judgements of facial attractiveness, age, gender, speech and expression. *Neuropsychologia* 35 685–693 10.1016/S0028-3932(96)00111-X 9153031

[B20] Butler S. Gilchrist I. D. Burt D. M. Perrett D. I. Jones E. Harvey M. (2005). Are the perceptual biases found in chimeric face processing reflected in eye-movement patterns? *Neuropsychologia* 43 52–59 10.1016/j.neuropsychologia.2004.06.005 15488905

[B21] Carey S. Diamond R. (1977). From piecemeal to configurational representation of faces. *Science* 195 312–314 10.1126/science.831281 831281

[B22] Chiandetti C. (2011). Pseudoneglect and embryonic light stimulation in the avian brain. *Behav. Neurosci.* 125 775–782 10.1037/a0024721 21859172

[B23] Chiang C. H. Ballantyne A. O. Trauner D. A. (2000). Development of perceptual asymmetry for free viewing of chimeric stimuli. *Brain Cogn.* 44 415–424 10.1006/brcg.1999.1202 11104534

[B24] Cochet H. Byrne R. W. (2013). Evolutionary origins of human handedness: evaluating contrasting hypotheses. *Anim. Cogn.* 16 531–542 10.1007/s10071-013-0626-y 23546932 PMC3684717

[B25] Coolican J. Eskes G. A. McMullen P. A. Lecky E. (2008). Perceptual biases in processing facial identity and emotion. *Brain Cogn.* 66 176–187 10.1016/j.bandc.2007.07.001 17720290

[B26] Corbetta M. Shulman G. L. (2002). Control of goal-directed and stimulus-driven attention in the brain. *Nat. Rev. Neurosci.* 3 201–215 10.1038/nrn755 11994752

[B27] Coren S. Porac C. (1977). Fifty centuries of right-handedness: the historical record. *Science* 198 631–632 10.1126/science.335510 335510

[B28] Dahl C. D. Rasch M. J. Tomonaga M. Adachi I. (2013). Laterality effect for faces in chimpanzees (*Pan troglodytes*). *J. Neurosci.* 33 13344–13349 10.1523/JNEUROSCI.0590-13.2013 23946392 PMC6705155

[B29] Dalby J. T. Gibson D. Grossi V. Schneider R. D. (1980). Lateralized hand gesture during speech. *J. Mot. Behav.* 12 292–297 10.1080/00222895.1980.10735228 15178538

[B30] Dellatolas G. Coutin T De Agostini M. (1996). Bisection and perception of horizontal lines in normal children. *Cortex* 32 705–715 10.1016/S0010-9452(96)80040-2 8954248

[B31] Diamond R. Carey S. (1977). Developmental changes in the representation of faces. *J. Exp. Child Psychol.* 23 1–22 10.1016/0022-0965(77)90069-8 839154

[B32] Diekamp B. Regolin L. Güntürkün O. Vallortigara G. (2005). A left-sided visuospatial bias in birds. *Curr. Biol.* 15 R372–R373 10.1016/j.cub.2005.05.017 15916935

[B33] Dundas E. M. Best C. A. Minshew N. J. Strauss M. S. (2012a). A lack of left visual field bias when individuals with autism process faces. *J. Autism Dev. Disord.* 42 1104–1111 10.1007/s10803-011-1354-2 21986874 PMC3428133

[B34] Dundas E. Gastgeb H. Strauss M. S. (2012b). Left visual field biases when infants process faces: a comparison of infants at high-and low-risk for autism spectrum disorder. *J. Autism. Dev. Disord.* 42 2659–2668 10.1007/s10803-012-1523-y 22527700 PMC3408549

[B35] Ellis H. D. Shepherd J. W. (1975). Recognition of upright and inverted faces presented in the left and right visual fields. *Cortex* 11 3–7 10.1016/S0010-9452(75)80014-1 1149464

[B36] Escalante-Mead P. R. Minshew N. J. Sweeney J. A. (2003). Abnormal brain lateralization in high-functioning autism. *J. Autism. Dev. Disord.* 33 539–543 10.1023/A:1025887713788 14594334

[B37] Fagard J. Lemoine C. (2006). The role of imitation in the stabilization of handedness during infancy. *J. Integr. Neurosci.* 5 519–533 10.1142/S0219635206001343 17245820

[B38] Failla C. V. Sheppard D. M. Bradshaw J. L. (2003). Age and responding-hand related changes in performance of neurologically normal subjects on the line-bisection and chimeric-faces tasks. *Brain Cogn.* 52 353–363 10.1016/S0278-2626(03)00181-7 12907180

[B39] Field A. P. (2006). Watch out for the beast: fear information and attentional bias in children. *J. Clin. Child Adolesc. Psychol.* 35 431–439 10.1207/s15374424jccp3503_8 16836480

[B40] Gardner M. R. Potts R. (2010). Hand dominance influences the processing of observed bodies. *Brain Cogn.* 73 35–40 10.1016/j.bandc.2010.02.002 20338681

[B41] Gallois P. Buquet C. Charlier J. Paris V. Hache J. C. Dereux J. F. (1989). Asymetrie dans l’activité perceptive visuelle des visages et des expressions faciales emotionnelles. *Rev. Neurol.* 145 661–664 2814162

[B42] Godard O. Fiori N. (2010). Sex differences in face processing: are women less lateralized and faster than men? *Brain Cogn.* 73 167–175 10.1016/j.bandc.2010.04.008 20621740

[B43] Golarai G. Ghahremani D. G. Whitfield-Gabrieli S. Reiss A. Eberhardt J. L. Gabrieli J. D. (2007). Differential development of high-level visual cortex correlates with category-specific recognition memory. *Nat. Neurosci.* 10 512–522 10.1038/nn1865 17351637 PMC3660101

[B44] Greene D. J. Zaidel E. (2011). Hemispheric differences in attentional orienting by social cues. *Neuropsychologia* 49 61–68 10.1016/j.neuropsychologia.2010.11.007 21093465

[B45] Greenough W. T. Black J. E. Wallace C. S. (1987). Experience and brain development. *Child Dev.* 58 539–559 10.2307/1130197 3038480

[B46] Guo K. Meints K. Hall C. Hall S. Mills D. (2009). Left gaze bias in humans, rhesus monkeys and domestic dogs. *Anim. Cogn.* 12 409–418 10.1007/s10071-008-0199-3 18925420

[B47] Hagemann N. (2009). The advantage of being left-handed in interactive sports. *Attent. Percept. Psychophys.* 71 1641–1648 10.3758/APP.71.7.1641 19801623

[B48] Harkins D. A. Michel G. F. (1988). Evidence for a maternal effect on infant hand – use preferences. *Dev. Psychobiol.* 21 535–541 10.1002/dev.420210604 3169378

[B49] Harkins D. A. Uẑgiris I. Č. (1991). Hand-use matching between mothers and infants during the first year. *Infant Behav. Dev.* 14 289–298 10.1016/0163-6383(91)90023-L

[B50] Heath R. Rouhana A Abi Ghanem D. (2005). Asymmetric bias in perception of facial affect among Roman and Arabic script readers. *Laterality* 10 51–64 10.1080/13576500342000293 15841823

[B51] Heller W. Levy J. (1981). Perception and expression of emotion in right-handers and left-handers. *Neuropsychologia* 19 263–272 10.1016/0028-3932(81)90110-X 7254505

[B52] Heller W. Etienne M. A. Miller G. A. (1995). Patterns of perceptual asymmetry in depression and anxiety: implications for neuropsychological models of emotion and psychopathology. *J. Abnorm. Psychol.* 104 327–333 10.1037/0021-843X.104.2.327 7790634

[B53] Heron-Delaney M. Wirth S. Pascalis O. (2011). Infants’ knowledge of their own species. *Philos. Trans. R. Soc. B Biol. Sci.* 366 1753–1763 10.1098/rstb.2010.0371 PMC3130380 21536558

[B54] Hobson R. P. Ouston J. Lee A. (1988). What’s in a face? The case of autism. *Br. J. Psychol.* 79 441–453 10.1111/j.2044-8295.1988.tb02745.x 3208000

[B55] Hopkins W. D. (2004). Laterality in maternal cradling and infant positional biases: implications for the development and evolution of hand preferences in nonhuman primates. *Int. J. Primatol.* 25 1243–1265 10.1023/B:IJOP.0000043961.89133.3d 18049716 PMC2099253

[B56] Hopkins W. D. (2006). Comparative and familial analysis of handedness in great apes. *Psychol. Bull.* 132 538–559 10.1037/0033-2909.132.4.538 16822166 PMC2063575

[B57] Hoptman M. Levy J. (1988). Perceptual asymmetries in left- and right-handers for cartoon and real faces. *Brain Cogn.* 8 178–188 10.1016/0278-2626(88)90048-6 3196482

[B58] Hsiao J. H. Cottrell G. (2008). Two fixations suffice in face recognition. *Psychol. Sci.* 19 998–1006 10.1111/j.1467-9280.2008.02191.x 19000210 PMC7360057

[B59] Huheey J. E. (1977). Concerning the origin of handedness in humans. *Behav. Genet.* 7 29–32 843315 10.1007/BF01067174

[B60] Indersmitten T. Gur R. C. (2003). Emotion processing in chimeric faces: hemispheric asymmetries in expression and recognition of emotions. *J. Neurosci.* 23 3820–3825 12736352 10.1523/JNEUROSCI.23-09-03820.2003 PMC6742199

[B61] Jaynes J. (1976). *The Origin of Consciousness in the Breakdown of the Bicameral Mind*. Boston: Houghton Mifflin

[B62] Jewell G. McCourt M. E. (2000). Pseudoneglect: a review and meta-analysis of performance factors in line bisection tasks. *Neuropsychologia* 38 93–110 10.1016/S0028-3932(99)00045-7 10617294

[B63] Kanwisher N. McDermott J. Chun M. M. (1997). The fusiform face area: a module in human extrastriate cortex specialized for face perception. *J. Neurosci.* 17 4302–4311 9151747 10.1523/JNEUROSCI.17-11-04302.1997 PMC6573547

[B64] Keller J. Nitschke J. B. Bhargava T. Deldin P. J. Gergen J. A. Miller G. A. (2000). Neuropsychological differentiation of depression and anxiety. *J. Abnorm. Psychol.* 109 3–10 10.1037/0021-843X.109.1.3 10740930

[B65] Kim H. Levine S. C. Kertesz S. (1990). Are variations among subjects in lateral asymmetry real individual differences or random error in measurement? Putting variability in its place. *Brain Cogn.* 14 220–242 10.1016/0278-2626(90)90031-I 2285515

[B66] Kimura D. (1973a). Manual activity during speaking – I. Right-handers.* Neuropsychologia* 11 45–50 10.1016/0028-3932(73)90063-8 4694780

[B67] Kimura D. (1973b). Manual activity during speaking–-II. Left-handers.* Neuropsychologia* 11 51–55 10.1016/0028-3932(73)90064-X 4694781

[B68] Kinsbourne M. (1970). The cerebral basis of lateral asymmetries in attention. *Acta Psychol.* 33 193–201 10.1016/0001-6918(70)90132-0 5445964

[B69] Klin A. Jones W. Schultz R. Volkmar F. (2003). The enactive mind, or from actions to cognition: lessons from autism. *Philos. Trans. R. Soc. Lond. B Biol. Sci.* 358 345–360 10.1098/rstb.2002.1202 12639332 PMC1693114

[B70] Kolb B. Wilson B. Taylor L. (1992). Developmental changes in the recognition and comprehension of facial expression: implications for frontal lobe function. *Brain Cogn.* 20 74–84 10.1016/0278-2626(92)90062-Q 1389123

[B71] Lavergne J. Kimura D. (1987). Hand movement asymmetry during speech: no effect of speaking topic. *Neuropsychologia* 25 689–693 10.1016/0028-3932(87)90060-1 3658152

[B72] Leehey S. Carey S. Diamond R. Cahn A. (1978). Upright and inverted faces: the right hemisphere knows the difference. *Cortex* 14 411–419 10.1016/S0010-9452(78)80067-7 710151

[B73] Leonards U. Scott-Samuel N. E. (2005). Idiosyncratic initiation of saccadic face exploration in humans. *Vision Res.* 45 2677–2684 10.1016/j.visres.2005.03.009 16042969

[B74] Levine S. C. Levy J. (1986). Perceptual asymmetry for chimeric faces across the life span. *Brain Cogn.* 5 291–306 10.1016/0278-2626(86)90033-3 3756006

[B75] Levine S. C. Banich M. T. Koch-Weser M. (1984). Variations in patterns of lateral asymmetry among dextrals. *Brain Cogn.* 3 317–334 10.1016/0278-2626(84)90024-1 6536331

[B76] Lindell A. K. (2013). Continuities in emotion lateralization in human and non-human primates. *Front. Hum. Neurosci.* 7:464. 10.3389/fnhum.2013.00464 PMC3737467 23964230

[B77] Liu S. Quinn P. C. Wheeler A. Xiao N. Ge L. Lee K. (2011). Similarity and difference in the processing of same-and other-race faces as revealed by eye tracking in 4-to 9-month-olds. *J. Exp. Child Psychol.* 108 180–189 10.1016/j.jecp.2010.06.008 20708745 PMC3740558

[B78] Luh K. E. (1998). Effect of inversion on perceptual biases for chimeric faces. *Brain Cogn.* 37 105–108

[B79] Luh K. E. Red J. Levy J. (1994). Left- and right-handers see people differently: free-vision perceptual asymmetries for chimeric stimuli. *Brain Cogn.* 14 141–160 10.1006/brcg.1994.1028 7917239

[B80] Marotta A. Lupiáñez J. Casagrande M. (2012). Investigating hemispheric lateralization of reflexive attention to gaze and arrow cues. *Brain Cogn.* 80 361–366 10.1016/j.bandc.2012.08.001 22959915

[B81] Marzoli D. Menditto S. Lucafò C. Tommasi L. (2013). Imagining others’ handedness: visual and motor processes in the attribution of the dominant hand to an imagined agent. *Exp. Brain Res.* 22 37–46 10.1007/s00221-013-3587-0 23743716

[B82] Marzoli D. Mitaritonna A. Moretto F. Carluccio P. Tommasi L. (2011a). The handedness of imagined bodies in action and the role of perspective taking. *Brain Cogn.* 75 51–59 10.1016/j.bandc.2010.10.002 21035936

[B83] Marzoli D. Palumbo R. Di Domenico A. Penolazzi B. Garganese P. Tommasi L. (2011b). The relation between self-reported empathy and motor identification with imagined agents. *PLoS ONE* 6:e14595 10.1371/journal.pone.0014595 PMC3027625 21298089

[B84] Maurer D. Le Grand R. Mondloch C. J. (2002). The many faces of configural processing. *Trends Cogn. Sci.* 6 255–260 10.1016/S1364-6613(02)01903-4 12039607

[B85] Megreya A. M. Havard C. (2011). Left face matching bias: right hemisphere dominance or scanning habits? *Laterality* 16 75–92 10.1080/13576500903213755 21204307

[B86] Meguerditchian A. Vauclair J. Hopkins W. D. (2013). On the origins of human handedness and language: a comparative review of hand preferences for bimanual coordinated actions and gestural communication in nonhuman primates. *Dev. Psychobiol.* 55 637–650 10.1002/dev.21150 23955015

[B87] Mertens I. Siegmund H. Grüsser O. J. (1993). Gaze motor asymmetries in the perception of faces during a memory task. *Neuropsychologia* 31 989–998 10.1016/0028-3932(93)90154-R 8232855

[B88] Mesulam M. (1981). A cortical network for directed attention and unilateral neglect. *Ann. Neurol.* 10 309–325 7032417 10.1002/ana.410100402

[B89] Michel G. F. (1992). Maternal influences on infant hand-use during play with toys. *Behav. Genet.* 22 163–176 10.1007/BF01066995 1596256

[B90] Mogg K. Bradley B. P. (1999). Orienting of attention to threatening facial expressions presented under conditions of restricted awareness. *Cogn. Emot.* 13 713–740 10.1080/026999399379050

[B91] Mogg K. Bradley B. P. (2002). Selective orienting of attention to masked threat faces in social anxiety. *Behav. Res. Ther.* 40 1403–1414 10.1016/S0005-7967(02)00017-7 12457635

[B92] Mondloch C. J. Le Grand R. Maurer D. (2002). Configural face processing develops more slowly than featural face processing. *Perception* 31 553–566 10.1068/p3339 12044096

[B93] Peelen M. V. Downing P. E. (2005). Selectivity for the human body in the fusiform gyrus. *J. Neurophysiol.* 93 603–608 10.1152/jn.00513.2004 15295012

[B94] Peelen M. V. Glaser B. Vuilleumier P. Eliez S. (2009). Differential development of selectivity for faces and bodies in the fusiform gyrus. *Dev. Sci.* 12 F16–F25 10.1111/j.1467-7687.2009.00916.x 19840035

[B95] Peelen M. V. Wiggett A. J. Downing P. E. (2006). Patterns of fMRI activity dissociate overlapping functional brain areas that respond to biological motion. *Neuron* 49 815–822 10.1016/j.neuron.2006.02.004 16543130

[B96] Peirce J. W. Leigh A. E. Kendrick K. M. (2000). Configurational coding, familiarity and the right hemisphere advantage for face recognition in sheep. *Neuropsychologia* 38 475–483 10.1016/S0028-3932(99)00088-3 10683397

[B97] Peirce J. W. Leigh A. E. daCosta A. P. C. Kendrick K. M. (2001). Human face recognition in sheep: lack of configurational coding and right hemisphere advantage. *Behav. Process.* 55 13–26 10.1016/S0376-6357(01)00158-9 11390088

[B98] Philip R. C. M. Whalley H. C. Stanfield A. C. Sprengelmeyer R. Santos I. M. Young A. W. (2010). Deficits in facial, body movement and vocal emotional processing in autism spectrum disorders. *Psychol. Med.* 40 1919–1929 10.1017/S0033291709992364 20102666

[B99] Prete G. D’Ascenzo S. Laeng B. Fabri M. Foschi N. Tommasi L. (2013). Conscious and unconscious processing of facial expressions: evidence from two split-brain patients. *J. Neuropsychol.* 10.1111/jnp.12034 [Epub ahead of print]. 24325712

[B100] Racca A. Guo K. Meints K. Mills D. S. (2012). Reading faces: differential lateral gaze bias in processing canine and human facial expressions in dogs and 4-year-old children. *PLoS ONE* 7:e36076. 10.1371/journal.pone.0036076 PMC3338636 22558335

[B101] Rahman Q. Anchassi T. (2012). Men appear more lateralized when noticing emotion in male faces. *Emotion* 12 174–179 10.1037/a0024416 21707155

[B102] Raymond M. Pontier D. Dufour A. B. Moller A. P. (1996). Frequency-dependent maintenance of left handedness in humans. *Proc. R. Soc. Lond. B Biol. Sci.* 263 1627–1633 10.1098/rspb.1996.0238 9025310

[B103] Reed C. L. Beall P. M. Stone V. E. Kopelioff L. Pulham D. J. Hepburn S. L. (2007). Brief report: perception of body posture – what individuals with autism spectrum disorder might be missing. *J. Autism. Dev. Disord.* 37 1576–1584 10.1007/s10803-006-0220-0 17029019

[B104] Reed C. L. Stone V. E. Bozova S. Tanaka J. (2003). The body-inversion effect. *Psychol. Sci.* 14 302–308 10.1111/1467-9280.14431 12807401

[B105] Reed C. L. Stone V. E. Grubb J. D. McGoldrick J. E. (2006). Turning configural processing upside down: part and whole body postures. *J. Exp. Psychol. Hum. Percept. Perform.* 32 73–87 10.1037/0096-1523.32.1.73 16478327

[B106] Reynolds D. M. Jeeves M. A. (1978). A developmental study of hemisphere specialization for recognition of faces in normal subjects. *Cortex* 14 511–520 10.1016/S0010-9452(78)80026-4 738061

[B107] Rosa Salva O. Regolin L. Vallortigara G. (2007). Chicks discriminate human gaze with their right hemisphere. *Behav. Brain Res.* 177 15–21 10.1016/j.bbr.2006.11.020 17174412

[B108] Roszkowski M. J. Snelbecker G. E. (1982). Validity and temporal stability of the chimeric face technique for studying hemispheric processing asymmetries: data from 6-through 14-year-old children. *J. Behav. Assess.* 4 209–221 10.1007/BF01321266

[B109] Sackeim H. A. Gur R. C. (1978). Lateral asymmetry in intensity of emotional expression. *Neuropsychologia* 16 473–481 10.1016/0028-3932(78)90070-2 692859

[B110] Sakhuja T. Gupta G. C. Singh M. Vaid J. (1996). Reading habits affect asymmetries in facial affect judgments: a replication. *Brain Cogn.* 32 162–165

[B111] Saucier D. M. Elias L. J. (2001). Lateral and sex differences in manual gesture during conversation. *Laterality* 6 239–245 10.1080/713754416 15513173

[B112] Scherf K. S. Behrmann M. Humphreys K. Luna B. (2007). Visual category-selectivity for faces, places and objects emerges along different developmental trajectories. *Dev. Sci.* 10 F15–F30 10.1111/j.1467-7687.2007.00595.x 17552930

[B113] Schwarzlose R. F. Baker C. I. Kanwisher N. (2005). Separate face and body selectivity on the fusiform gyrus. *J. Neurosci.* 25 11055–11059 10.1523/JNEUROSCI.2621-05.2005 16306418 PMC6725864

[B114] Scola C. Vauclair J. (2010). Is infant holding-side bias related to motor asymmetries in mother and child? *Dev. Psychobiol.* 52 475–486 10.1002/dev.20450 20583144

[B115] Slaughter V. Heron M. Sim S. (2002). Development of preferences for the human body shape in infancy. *Cognition* 85 B71–B81 10.1016/S0010-0277(02)00111-7 12169414

[B116] Slaughter V. Stone V. E. Reed C. (2004). Perception of faces and bodies similar or different? *Curr. Dir. Psychol. Sci.* 13 219–223 10.1111/j.0963-7214.2004.00312.x

[B117] Soria Bauser D. A. Suchan B. Daum I. (2011). Differences between perception of human faces and body shapes: evidence from the composite illusion. *Vision Res.* 51 195–202 10.1016/j.visres.2010.11.007 21093471

[B118] Tamietto M. Castelli L. Vighetti S. Perozzo P. Geminiani G. Weiskrantz L de Gelder B. (2009). Unseen facial and bodily expressions trigger fast emotional reactions. *Proc. Natl. Acad. Sci. U.S.A.* 106 17661–17666 10.1073/pnas.0908994106 19805044 PMC2764895

[B119] Tantam D. Monaghan L. Nicholson H. Stirling J. (1989). Autistic children’s ability to interpret faces: a research note. *J. Child Psychol. Psychiatry* 30 623–630 10.1111/j.1469-7610.1989.tb00274.x 2768363

[B120] Taylor S. Workman L. Yeomans H. (2012). Abnormal patterns of cerebral lateralisation as revealed by the universal chimeric faces task in individuals with autistic disorder. *Laterality* 17 428–437 10.1080/1357650X.2010.521751 22690895

[B121] Thompson L. A. Malloy D. M. LeBlanc K. L. (2009). Lateralization of visuospatial attention across face regions varies with emotional prosody. *Brain Cogn.* 69 108–115 10.1016/j.bandc.2008.06.002 18639372 PMC2698616

[B122] Tonks J. Williams W. H. Frampton I. Yates P. Slater A. (2007). Assessing emotion recognition in 9-15-years olds: preliminary analysis of abilities in reading emotion from faces, voices and eyes. *Brain Injury* 21 623–629 10.1080/02699050701426865 17577713

[B123] Tsai L. Y. (1982). Brief report: handedness in autistic children and their families. *J. Autism. Dev. Disord.* 12 421–423 10.1007/BF01538328 7161240

[B124] Vaid J. Singh M. (1989). Asymmetries in the perception of facial affect: is there an influence of reading habits. *Neuropsychologia* 27 1277–1287 10.1016/0028-3932(89)90040-7 2594174

[B125] Vallortigara G. Rogers L. J. (2005). Survival with an asymmetrical brain: advantages and disadvantages of cerebral lateralization. *Behav. Brain Sci.* 28 575–589 10.1017/S0140525X05000105 16209828

[B126] van de Riet W. A de Gelder B. (2008). Watch the face and look at the body! *Neth. J. Psychol.* 64 143–151 10.1007/BF03076417

[B127] Vervloed M. P. J. Hendriks A. W van den Eijnde E. (2011). The effects of mothers’ past infant-holding preferences on their adult children’s face processing lateralisation. *Brain Cogn.* 75 248–254 10.1016/j.bandc.2011.01.002 21272982

[B128] Voelz Z. R. Gencoz F. Gencoz T. Pettit J. W. Perez M. Joiner T. E. Jr (2001). Patterns of hemispheric perceptual asymmetries: left hemispatial biases predict changes in anxiety and positive affect in undergraduate women. *Emotion* 1 339–347 10.1037/1528-3542.1.4.339 12901396

[B129] Watling D. Bourne V. J. (2013). Sex differences in the relationship between children’s emotional expression discrimination and their developing hemispheric lateralization. *Dev. Neuropsychol.* 38 496–506 10.1080/87565641.2013.826660 24138218

[B130] Watling D. Workman L. Bourne V. J. (2012). Emotion lateralisation: developments throughout the lifespan. *Laterality* 17 389–411 10.1080/1357650X.2012.682160 22690893

[B131] Westergaard G. C. Suomi S. J. (1996). Hand preference for a bimanual task in tufted capuchins (*Cebus apella*) and rhesus macaques (*Macaca mulatta*). *J. Comp. Psychol.* 110 406–411 10.1037/0735-7036.110.4.406 8956511

[B132] Wheeler A. (2010). *The Emergence of a Left Visual Field Bias in Infants’ Processing of Dynamic Faces*. Doctoral dissertation, University of Toronto Toronto

[B133] Willems R. M. Peelen M. V. Hagoort P. (2010). Cerebral lateralization of face-selective and body-selective visual areas depends on handedness. *Cereb. Cortex* 20 1719–1725 10.1093/cercor/bhp234 19889713

[B134] Williams J. H. Whiten A. Singh T. (2004). A systematic review of action imitation in autistic spectrum disorder. *J. Autism. Dev. Disord.* 34 285–299 10.1023/B:JADD.0000029551.56735.3a 15264497

[B135] Workman L. Chilvers L. Yeomans H. Taylor S. (2006). Development of cerebral lateralisation for recognition of emotions in chimeric faces in children aged 5 to 11. *Laterality* 11 493–507 10.1080/13576500600724963 16966239

[B136] Workman L. Peters S. Taylor S. (2000). Lateralisation of perceptual processing of pro-and anti-social emotions displayed in chimeric faces. *Laterality* 5 237–249 10.1080/713754378 15513144

[B137] Young A. W. Bion P. J. (1980). Absence of any developmental trend in right hemisphere superiority for face recognition. *Cortex* 16 213–221 10.1016/S0010-9452(80)80057-8 7471762

[B138] Young A. W. Ellis H. D. (1976). An experimental investigation of developmental differences in ability to recognize faces presented to the left and right cerebral hemispheres. *Neuropsychologia* 14 495–498 10.1016/0028-3932(76)90078-6 995242

[B139] Yovel G. Pelc T. Lubetzky I. (2010). It’s all in your head: why is the body inversion effect abolished for headless bodies? *J. Exp. Psychol. Hum. Percept. Perform.* 36 759–767 10.1037/a0017451 20515202

[B140] Yovel G. Tambini A. Brandman T. (2008). The asymmetry of the fusiform face area is a stable individual characteristic that underlies the left-visual-field superiority for faces. *Neuropsychologia* 46 3061–3068 10.1016/j.neuropsychologia.2008.06.017 18639566

[B141] Zartor A. S. Mikheev M. M. Afanasiev S. V. (2010). Lateral peculiarities of the process of mental rotation of the human body scheme. *J. Evol. Biochem. Physiol.* 46 292–294 10.1134/S0022093010030117 20583588

